# Editorial: Dysbiosis, obesity, and inflammation: interrelated phenomena causes or effects of metabolic syndrome?

**DOI:** 10.3389/fendo.2023.1265314

**Published:** 2023-10-17

**Authors:** Kaiser Wani, Shakilur Rahman, Hossam Draz

**Affiliations:** ^1^ Chair for Biomarkers of Chronic Diseases, Biochemistry Department, College of Science, King Saud University, Riyadh, Saudi Arabia; ^2^ University Institute of Biotechnology, Chandigarh University, Mohali, India; ^3^ Department of Medical Elementology and Toxicology, School of Chemical and Life Sciences, Jamia Hamdard, New Delhi, India; ^4^ Department of Anesthesiology and Perioperative Medicine, University of Alabama at Birmingham, Birmingham, AL, United States; ^5^ Charles River Laboratories, Senneville, QC, Canada

**Keywords:** gut microbiota, dysbiosis, obesity, metabolic syndrome, inflammation, metabolically healthy obese

## Introduction

The gut microbiome, a diverse community of microorganisms in the gastrointestinal tract, plays a crucial role in metabolism and is associated with various metabolic disorders. Extensive evidence suggests that the composition of the gut microbiome is associated with various metabolic disorders (1, 2). However, there is no consensus on the causal link between gut microbiome and type 2 diabetes mellitus (T2DM), and the specific taxonomic groups responsible for T2DM remain unclear. Dysbiosis, referring to an imbalance or disruption in the gut microbiota composition, leads to increased levels of lipopolysaccharide (LPS), a bacterial toxin, in the body (3). LPS is a potent endotoxin present in the outer membrane of Gram-negative bacteria. It is a key player in inducing inflammation by activating the toll-like receptor 4 (TLR4) pathway, leading to the release of pro-inflammatory cytokines and chemokines that initiate and propagate the inflammatory response (4). Recent studies have highlighted the critical role of LPS-induced inflammation in various diseases, including sepsis, inflammatory bowel disease, and chronic inflammatory conditions (5).

## Summary of articles published

In this Research Topic, we focused on the interactions between dysbiosis, obesity, inflammation, and metabolic parameters. The most common microvascular complication caused by inflammatory stress associated with metabolic disorders like diabetes mellitus is diabetic retinopathy (DR). Recent studies suggest that the gut microbiota is crucial to the development of DR and is implicated in its pathophysiological processes (6, 7). On the one hand, several studies have shown how the gut bacteria contribute to retinal neurodegeneration (8) while on the other hand, some studies show that altered gut flora in these patients may contribute to or aggravate DR (9). A review published under this study topic by Zhang and Mo tries to emphasize the significant connection between DR and gut microbiota and the importance of gut dysbiosis in the emergence and progression of DR. Furthermore, it explores the concept of the gut-retina axis and the conditions of the gut-retina axis crosstalk, along with the process involved in modulating DR by the intestinal microbiota.

Metabolic disorders like obesity are associated with risks of developing gastrointestinal (GI) disease (10). High fat diet-induced obesity in mice promotes dysbiosis, causing a shift towards bacteria-derived metabolites which contributes to the onset and progression of GI disorders (11). Moreover, there are two categories of obesity: metabolically healthy and metabolically unhealthy. In contrast to metabolically healthy counterparts, obese individuals who are metabolically unhealthy display hallmark symptoms of the metabolic syndrome (e.g., hypertension, dyslipidemia, hyperglycemia, abdominal obesity) (12). Obesity is often also accompanied by gastroesophageal reflux disease (GERD) (13). Due to their widespread availability, proton-pump inhibitors (PPIs) are most frequently used to treat heartburn and other related symptoms associated with GERD (14). A review article published in this Research Topic by Burmeister et al. discuss how a poor diet, along with both short-term and long-term PPI usage, negatively impacts the GI microbiota to cause dysbiosis. Furthermore, this article also covers the advantage of taking probiotics to mitigate PPI-induced dysbiosis and metabolically unhealthy obesity (MUO).

High plasma triglyceride levels and chronic inflammation are important factors in metabolic-associated fatty liver disease (15, 16). Elevated triglyceride levels contribute to the accumulation of fat in the liver, while chronic inflammation exacerbates liver damage and promotes the transformation of fatty liver to more severe stages of the disease (17). Moreno-Vedia et al. in a study published under this topic used nuclear magnetic resonance (1H-NMR) to examine triglyceride-rich lipoprotein (TLR) and glycoprotein profiles in 280 patients with metabolic disease. TRL concentrations were associated with glycoproteins and liver function. Follow-up revealed new cases of fatty liver associated with baseline TRL particle numbers and glycoprotein levels. Higher TRL levels were observed in patients with hepatic steatosis, and baseline TRL particles and glycoproteins were associated with the development of metabolic-associated fatty liver disease (MAFLD). The findings suggest that TLR measurements could serve as predictive biomarkers for hepatic disease.

An article by Wang et al., published under this Research Topic, reviewed the effects of oral glucose-lowering drugs on gut microbiota and microbial metabolites. Increasing evidence suggests that oral glucose-lowering drugs modulate the gut microbiome and alter GI metabolites. Antidiabetic medication such as metformin and sulfonylurea modify the intestinal flora in T2DM in clinical research and experimental animal studies (18–20). This review also highlights the future perspective of these drugs, such as combination therapies including pre- and pro-biotics intervention in T2DM. Another study under this Research Topic explored the associations between gut microbiota and glucose metabolism in a cohort of African and Chinese healthy individuals (Nizigiyimana et al.). Microbiota diversity, richness, and composition were higher in the African group and lower in the Chinese group. The phylum *Bacteroidetes* was significantly more abundant in the Chinese group. In contrast, the phylum *Verrucomicrobia* was significantly more prevalent in the African group. Gut microbiota also correlated with parameters of glucose metabolism. The data suggest that there is an interaction between gut microbiota, and glucometabolic pathways.

Probiotic administration significantly reduces faecal and plasma concentrations of LPS in patients by reducing LPS producing bacteria and related synthesis pathways (21). Probiotics, such as *Lactobacillus* and *Bifidobacterium*, protect the gut barrier by enhancing the expression of tight junction proteins and reducing inflammation (22, 23). This concept was utilized in a randomized clinical trial by Lin et al., published under this topic, where probiotics were administered to assess their effects on alleviating postoperative complications from thyroid hormone withdrawal (THW) in thyroid cancer patients. Probiotics showed promising results in reducing complications, including lack of energy, constipation, weight gain, and dry mouth, and improving lipid indicators. They also restored gut and oral microbial diversity by increasing beneficial bacteria and reducing harmful ones. This study thus highlighted the potential of probiotics in managing THW-related complications through microbiota modulation.

Palmitoylethanolamide (PEA) is an endogenous lipid mediator that exerts anti-inflammatory effects by targeting various pathways involved in inflammation. It interacts with peroxisome proliferator-activated receptors (PPARs), leading to the downregulation of pro-inflammatory genes and the upregulation of anti-inflammatory genes (24). Additionally, PEA can inhibit immune cell recruitment promoting the synthesis of serotonin and other anti-inflammatory compounds, which collectively contribute to its anti-inflammatory properties (25). In this study topic, an article published by Pirozzi et al. also showed that PEA reduced intestinal immune cell recruitment, inflammatory response triggered by high fat diet, and corticotropin releasing hormone levels. It suggested that PEA also altered tryptophan metabolism and promotes serotonin synthesis through increased butyrate-producing bacteria, such as *Bifidobacterium, Oscillospiraceae and Turicibacter sanguinis*.

Bilirubin, a byproduct of heme metabolism, has various metabolic advantages (26–28). The link between bilirubin and metabolically healthy obesity (MHO), however, is not frequently documented. The article published here by Fu et al. elucidates the associations between serum bilirubin levels and metabolic parameters in different obesity phenotypes. For this, amongst 1,042 participants, 541 were obese patients and 501 were healthy control subjects. The obese patients were further divided into MHO group and metabolically unhealthy obesity (MUHO) group according to the levels of fasting plasma glucose (FBG), triglyceride (TG), high-density lipoprotein cholesterol (HDL-C) and blood pressure (BP). It was observed that compared with MUHO group, MHO group had favorable BP, glucose and lipid profiles, apart from increased total bilirubin (TBil) and direct bilirubin (DBil) levels, and decreased hsCRP and HOMA-IR levels. Multivariate regression analysis shows that HOMA-IR is independently correlated with TBil and DBil levels.

## Conclusion

This editorial focuses on findings published in the Research Topic “*Dysbiosis, Obesity, and Inflammation: Interrelated Phenomena Causes or Effects of Metabolic Syndrome?*”. Recent evidence supports the significant role of gut microbiota in diabetic retinopathy and other metabolic complications. Additionally, it discusses the therapeutic potential of probiotics and endogenous lipid mediator, palmitoylethanolamide, in reducing inflammation and managing metabolic diseases. Moreover, it explores the associations between elevated triglyceride levels, chronic inflammation, and metabolic-associated fatty liver disease. Overall, this Research Topic provides valuable insights into the gut microbiota’s impact on metabolic health and potential interventions for metabolic disorders.

## Author contributions

KW: Conceptualization, Writing – original draft, Writing – review & editing. SR: Writing – original draft, Writing – review & editing. HD: Writing – original draft, Writing – review & editing.
